# Development of an RPA-CRISPR/Cas12a Assay for Rapid and Sensitive Diagnosis of Plant Quarantine Fungus *Setophoma terrestris*

**DOI:** 10.3390/jof10100716

**Published:** 2024-10-15

**Authors:** Peng Zhao, Zhipeng Feng, Lei Cai, Dorji Phurbu, Weijun Duan, Fuhong Xie, Xuelian Li, Fang Liu

**Affiliations:** 1 State Key Laboratory of Mycology, Institute of Microbiology, Chinese Academy of Sciences, Beijing 100101, China; 2 College of Life Sciences, University of Chinese Academy of Sciences, Beijing 100049, China; 3 Tibet Key Laboratory of Plateau Fungi, Lhasa 850000, China; 4 Tibet Plateau Institute of Biology, Lhasa 850000, China; 5 Ningbo Academy of Inspection and Quarantine, Ningbo 315012, China; 6 Technical Center of Ningbo Customs District P.R. China, Ningbo 315012, China; 7 Henan Engineering Research Center of Industrial Enzymes, Biology Institute of Henan Academy of Sciences, Zhengzhou 450008, China

**Keywords:** fluorescence detection, lateral flow detection, onion pink root rot, phytosanitary pathogenic fungi, RPA-CRISPR/Cas12a, visual detection

## Abstract

*Setophoma terrestris* is an important phytopathogenic fungus listed by China as a harmful fungus subject to phytosanitary import control. This pathogen is a threat to a wide range of plants, particularly as the causal agent of onion pink root rot, one of the most severe diseases of onions. In order to provide rapid identification and early warning of *S. terrestris* and prevent its spread, we have developed a rapid, accurate, and visually intuitive diagnostic assay for this pathogen, by utilizing recombinase polymerase amplification (RPA), coupled with CRISPR/Cas12a cleavage and fluorescence-based detection systems or paper-based lateral flow strips. The developed RPA-CRISPR/Cas12a assay exhibited remarkable specificity for the detection of *S. terrestris.* Moreover, this protocol can detect the pathogen at a sensitivity level of 0.01 pg/μL, which significantly outperforms the 1 pg/μL sensitivity achieved by the existing qPCR-based detection method. The entire diagnostic procedure, including DNA extraction, the RPA reaction, the Cas12a cleavage, and the result interpretation, can be accomplished in 40 min. Furthermore, the successful application of the assay in infected plant samples highlighted its potential for rapid and accurate pathogen detection in agricultural settings. In summary, this RPA-CRISPR/Cas12a diagnostic method offers a potentially valuable technological solution for quarantine and disease management.

## 1. Introduction

*Setophoma terrestris* (H.N. Hansen) Gruyter (syn. *Pyrenochaeta terrestris* (H.N. Hansen) Gorenz, *Phoma terrestris* H.N. Hansen) is an important plant pathogenic fungus that can infect various plants, including *Allium cepa*, *A. sativum*, *A. porrum*, *Avena sativa*, *Calathea crocata*, *Cucurbita maxima*, *C. moschata*, *Medicago sativa*, *Oryza sativa*, *Phaseolus lunatus*, *Pisum sativum*, *Solanum lycopersicum*, *Triticum aestivum*, *Vigna sinensis*, *Zea mays* [1,2,3,4,5,6,7,8]. It has been reported in countries including Argentina, Australia, Brazil, Canada, India, Japan, Mexico, Senegal, South Africa, the Netherlands, the United States, Venezuela, and Vietnam. Additionally, there have been reports in the Shandong and Jiangsu provinces of China [9].

Onions are widely grown in China and are one of China’s important export vegetables. According to statistics, China’s total onion production in 2020 accounted for 22.69% of the world’s total production. The pink root rot of onions caused by *S. terrestris* is one of the most severe diseases induced by this pathogen. Infected onion roots first show a light pink color that progresses to shades of red and purple, eventually shriveling and blackening, which results in poor growth of the onions [3,7]. The pinkish-red discoloration may extend into the bulb scales, and prolonged infection can lead to stunted plant growth. Typically, the infection is limited to the roots and outer bulb scales. Due to the severe threat posed by this pathogen and the significant economic losses that may be caused by its introduction into China, it has been included in the Chinese quarantine pest list by the Ministry of Agriculture and Rural Affairs of the People’s Republic of China (http://dzs.customs.gov.cn/dzs/2746776/3699554/index.html; accessed 15 May 2021).

With increasing international trade, the possibility of the introduction and establishment of this pathogen in non-endemic areas is considerable. Early warning, rapid detection, risk analysis, and scientific control of invasive species are vital for preventing the spread of exotic organisms. Among these, the accurate identification of *S. terrestris* plays a key role in controlling its damage, halting its transmission and spread in a timely manner. Therefore, establishing a specific, sensitive, rapid, and effective molecular detection method for the pathogen is of great significance for facilitating rapid clearance of imported plants and plant products and early field diagnosis of diseases.

In the past, the identification of *S. terrestris* was mainly based on morphological characteristics and phylogenetic analyses [8,10], which require a long time and well-trained staff. As an alternative approach, a recently developed real-time PCR approach provides fast and accurate results as compared to culture-based methods [11]. However, real-time PCR requires high technical expertise from operators and expensive laboratory equipment, making on-site testing challenging. In comparison, recombinase polymerase amplification (RPA), a nucleic acid assay considered to be an alternative to PCR, can be performed at a constant temperature of 37–42 °C, and the time can be controlled to less than 30 min. Therefore, RPA is a suitable amplification method for portable rapid nucleic acid detection on-site.

Furthermore, the combined utilization of RPA with clustered regularly interspaced short palindromic repeats (CRISPR)-based detection technology demonstrates superior accuracy, rapidity, sensitivity, and convenience in the identification of various pathogenic microbes when compared to traditional methodologies [12]. The foundation of this detection approach lies in the collateral nuclease activity of CRISPR-associated proteins such as Cas12 and Cas13, which recognize target nucleic acid sequences while simultaneously cleaving single-stranded DNA (ssDNA) reporter probes [13]. Specifically, the CRISPR/Cas12a system, guided by a 41 to 44 nt single CRISPR RNA (crRNA), exhibits precise recognition and cleavage of double-stranded DNA, accompanied by the collateral cleavage of a reporter probe, thus representing a novel generation of rapid and accurate nucleotide acid detection techniques [14]. Additionally, two prevalent signal reporter systems frequently integrated into CRISPR/Cas12a-based methodologies are paper-based lateral flow strips (PLFS) and fluorescence-based detection systems (FRB). The PLFS detection mechanism, complemented by colloidal gold nanoparticles, delivers expeditious and straightforward results within a 10 min timeframe for positive reactions, while the FRB detection method generates detectable fluorescence visible to the naked eye under blue light. This innovative platform has been effectively utilized across a few pathogenic fungi, such as diagnosing *Diaporthe aspalathi*, *Diaporthe caulivora*, *Fusarium verticillioides* through RPA-CRISPR/Cas12a combined with a lateral flow assay [15,16], and detecting *Verticillium dahliae* by uniting RPA-CRISPR/Cas12a with fluorescence-based detection systems [17].

In this study, we introduce a method for the rapid on-site detection of *S. terrestris* using the CRISPR/Cas12a system. This strategy entails a combination of RPA reaction, Cas12a cleavage post-recognition of the target, and visualization through PLFS and FRB.

## 2. Materials and Methods

### 2.1. Sample Collection

The DNA of two *S. terrestris* strains, CBS 335.29 and CBS 122483, was obtained from the culture collection (CBS) of the Westerdijk Fungal Biodiversity Institute in the Netherlands and used for molecular diagnosis. Seven closely related strains of *Setophoma* were accumulated from our previous work (Table 1), which were preserved in the LC culture collection (a personal culture collection of Lei Cai, housed in the Institute of Microbiology, Chinese Academy of Sciences). All strains used in this study were accurately identified based on morphological characteristics and multi-locus (ITS, *gapdh*, *tef-1α*, *tub2*) phylogenetic analysis in previous studies [8,10,18].

### 2.2. DNA Extraction

The strains were grown at 25 °C for 7 days, and the hyphae were scraped with a scalpel into the 2 mL centrifuge tube. For routine genomic DNA extraction, we used a modified CTAB protocol [19]. DNA concentration was quantified using NanoDrop™One (Thermo Fisher Scientific, Waltham, MA, USA).

### 2.3. Design of Primers and crRNA

**Figure 1:**
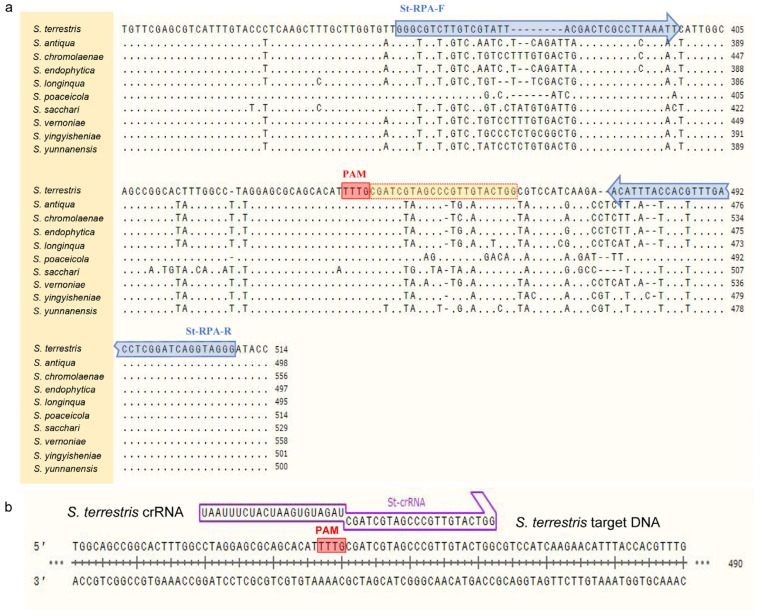
Alignment of ITS sequences of *Setophoma* species. (**a**) Location of RPA primers (blue arrows) and crRNA binding sites (in yellow) recognized by PAM region (in red) in ITS region of *S. terrestris* were labeled. The direction of blue arrow indicates the direction of the RPA primers. (**b**) Detailed schematic of how *S. terrestris* crRNA and target gene fragments bind.

ITS sequences of *Setophoma* strains listed in Table 1 were obtained from the NCBI GenBank and aligned using MAFFT v. 7 “http://mafft.cbrc.jp/alignment/server/index.html (accessed on 10 May 2023)”. After that, the conservative region of *S. terrestris* and the variant region for other species were chosen to design primer pairs St-RPA-F/St-RPA-R according to the manual of TwistAmp^®^ DNA Amplification Kits (Figure 1 and Table 2). It is worth noting that, when determining target sequences and designing primers, the amplicon contained at least one protospacer adjacent motif (PAM) site (5′-TTTN-3′) to facilitate recognition by Cas12a [14].

For Cas12a cleavage, the crRNA used in the CRISPR detection system was designed based on the amplified product from *S. terrestris* (Figure 1) and synthesized by Sangon Biotech. The FAM/BHQ1 labeled single-stranded DNA (ssDNA) was employed and cleaved for the RPA-Cas12a-mediated real-time and end-point fluorescence assay. In contrast, the FAM/Biotin labeled ssDNA was employed for lateral flow strip detection. The complete sequence of crRNA included a direct repeat sequence for Cas12a recognition 5′-UAAUUUCUACUAAGUGUAGAU-3′ (scaffold sequence) and a spacer sequence 5′-CGAUCGUAGCCCGUUGUACUGG-3′ (guide sequence) (Figure 1).

### 2.4. RPA Reaction

The RPA reaction was prepared using the TwistAmp™ Basic Kit (TwistDx™, Cambridge, UK). The RPA was generated by adding 29.5 μL rehydration buffer, 11.2 μL DEPC-H_2_O, 2.4 μL St-RPA-F primer (10 μmol/L), 2.4 μL St-RPA-R primer (10 μmol/L), 2 μL extracted DNA, and 2.5 μL magnesium acetate (280 mmol/L) to preconfigured reaction tubes containing the recombinase, polymerase, and single-strand binding protein. The mixture was then incubated at 39 °C for 30 min using a T30D tri-block super-gradient PCR system (LongGene, Hangzhou, Zhejiang, China). Then, the amplification products were detected by agarose gel electrophoresis.

To optimize the RPA reaction condition, the other parameters were kept unchanged, and the RPA time (set to 10, 20, 25, 30, 40 min) was adjusted to select the optimal reaction time based on the band brightness. After that, using the selected time and keeping other parameters unchanged, RPA temperature (set to 33, 35, 37, 39 and 41 °C) was adjusted to select the optimized temperature based on the band brightness.

### 2.5. CRISPR/Cas12a-FRB Visual Detection

The RPA-CRISPR/Cas12a-FRB assay system contains 2 μL of the above RPA product, 14.4 μL DEPC-H_2_O, 2 μL NEBuffer (10×), 0.4 μL Cas12a (5 μmol/L, New England Biolabs, Ipswich, MA, USA), 0.4 μL St-crRNA (0.01 mmol/L), 0.8 μL FQ-DNA reporter (5 μmol/L). After incubating under 37 °C for 30 min to perform CRISPR/Cas12a cleavage assay, the products were detected directly by the naked eye under blue light. Positive reactions were indicated by visible green fluorescent light, whereas negative reaction systems were transparent and colorless.

Next, to optimize the reaction conditions of the CRISPR/Cas12a-FRB detection system, the concentration ratio of Cas12a to crRNA was systematically adjusted (0 nM/0 nM, 50 nM/100 nM, 100 nM/200 nM, 150 nM/250 nM, 200 nM/300 nM), with subsequent observation of the fluorescence signal intensity under each condition. Subsequently, the concentration of the fluorescent reporter was fine-tuned, with the FQ-DNA concentrations set at 50 nmol/L, 100 nmol/L, 200 nmol/L, 400 nmol/L, and 800 nmol/L, while maintaining other parameters constant. Finally, the reaction time was optimized by varying it among incubation of 5 min, 10 min, 15 min, 20 min, and 30 min, followed by the assessment of fluorescence signal intensity under the diverse time conditions.

Using the optimized reaction conditions obtained above, the sensitivity of the RPA-CRISPR/Cas12a-FRB assay was further evaluated. DNA was serially diluted 10-fold to concentrations of 1 ng/μL, 0.1 ng/μL, 10 pg/μL, 1 pg/μL, 0.1 pg/μL, and 0.01 pg/μL. Each sample was tested in triplicate with sterile water as the negative control.

### 2.6. CRISPR/Cas12a-PLFS Visual Detection

**Figure 2:**
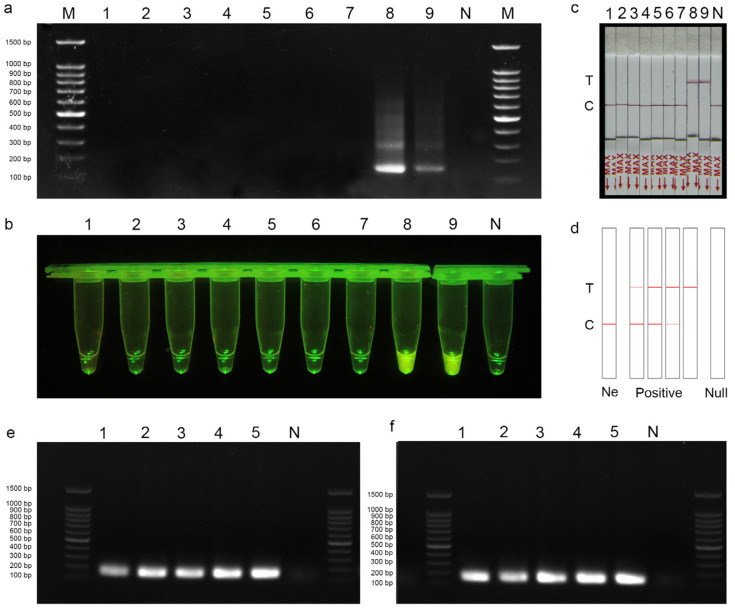
(**a**–**d**) Specificity of RPA and RPA-CRISPR/Cas12a primer for *S. terrestris.* (**a**) RPA detection assay. (**b**) RPA-CRISPR/Cas12a-FRB. (**c**) RPA-CRISPR/Cas12a-PLFS assay. (**d**) Schematic diagram of test strip test results, T: test band, C: control band, Ne: negative. M: 100 bp DNA ladder; 1–9: *S. endophytica*, *S. endophytica*, *S. antiqua*, *S. longinqua*, *S. yunnanensis*, *S. caverna*, *S. yingyisheniae*, *S. terrestris*, *S. terrestris*; N: ddH_2_O. (**e**) Optimization of RPA reaction time. 1–5: 10 min, 20 min, 25 min, 30 min, 40 min; N: ddH_2_O. (**f**) Optimization of RPA reaction temperature. 1–5: 33 °C, 35 °C, 37 °C, 39 °C, 41 °C; N: ddH_2_O.

The RPA-CRISPR/Cas12a-PLFS assay system contains 2 μL RPA product, 13.2 μL DEPC-H_2_O, 2 μL NEBuffer (10×), 0.4 μL Cas12a (5 μmol/L, New England Biolabs, Ipswich, MA, USA), 0.4 μL St-crRNA (0.01 mmol/L), 2 μL LF-DNA reporter (5 μmol/L). After incubating under 37 °C for 20 min to perform CRISPR/Cas12a cleavage assay, 80 μL DEPC-H_2_O was added to the reaction tube and mixed well. Then, the lateral flow strip (Suzhou Gendx Biotech Co., Ltd., Suzhou, China) was inserted into the tube and incubated at room temperature for 7 min. A positive result is indicated by the presence of a red band at the test line or at both the test line and control line on the strip. Conversely, a negative result is indicated by the absence of a red band at the test line but the presence of a red band at the control line (Figure 2d).

Subsequently, to optimize the reaction conditions of the CRISPR/Cas12a-PLFS system, the concentration of the reporter in the 100 μL reaction system was fine-tuned, with the LF-DNA concentrations set at 50 nmol/L, 100 nmol/L, 200 nmol/L, 400 nmol/L, and 800 nmol/L, while maintaining other parameters constant. Subsequently, the reaction time was optimized by varying it among incubation of 5 min, 10 min, 15 min, 20 min, and 30 min.

Using the optimized reaction conditions obtained above, the sensitivity of the RPA-CRISPR/Cas12a-PLFS assay was further evaluated. DNA was serially diluted 10-fold to concentrations of 1 ng/μL, 0.1 ng/μL, 10 pg/μL, 1 pg/μL, 0.1 pg/μL, and 0.01 pg/μL. Each sample was tested in triplicate with sterile water as the negative control.

### 2.7. Application of RPA-CRISPR/Cas12a Assay for Plant Samples

Application of the RPA-CRISPR/Cas12a assay for plant samples was assessed using inoculated and healthy onions. To obtain the plant samples, the strain for inoculation and the onion samples to be inoculated should be prepared at the same time. Seventeen purchased healthy onions were hydroponically grown, and the pathogen inoculation test was carried out after the growth of fibrous roots. Strain CBS 335.29 was cultured on PDA medium for 15 d. Agar blocks (5 mm in diameter) carrying mycelia were then cut from PDA plates and placed on the fibrous roots of twelve onions that were stabbed with a sterile inoculation needle. Five onions inoculated with sterile agar blocks were used as control. The onions were then placed in 9 cm Petri dishes with four layers of moisturizing gauze at the bottom and incubated at 25 °C. After 7 d of inoculation, one fibrous root sample from each onion was cut from the inoculated and control groups. The DNA of each root sample was extracted separately as the detection template using a modified CTAB protocol [19], and the genomic DNA of strain CBS 335.29 was used as the positive control, and sterile water instead of template was used as the negative control for the RPA-CRISPR/Cas12a assay in order to evaluate the effectiveness of the detection methods. The optimized reaction conditions of RPA-CRISPR/Cas12a-FRB and RPA-CRISPR/Cas12a-PLFS were used for the detection of plant samples.

## 3. Results

### 3.1. Specificity of RPA and RPA-CRISPR/Cas12a Primers and crRNA

To evaluate the specificity of the designed primer pair St-RPA-F/St-RPA-R, the RPA amplification was carried out using the strains in Table 1. Agarose gel electrophoresis results showed that *S. terrestris* samples showed obvious target bands, and the total length of the amplified product was 145 bp, while other fungal species did not show amplified bands (Figure 2a). After selecting the detection primer pairs, one of the PAM sites (5′-TTTG-3′) in the amplicon was chosen, and the following sequence was selected as the target sequence of crRNA for *S. terrestris* detection (Figure 1b).

The specificity of RPA-CRISPR/Cas12a-FRB and RPA-CRISPR/Cas12a-PLFS assay for *S. terristris* was subsequently evaluated using the species documented in Table 1. The detection results of the RPA-CRISPR/Cas12a-FRB assay demonstrated distinct fluorescent signals in the amplified products of the *S. terrestris* samples, whereas no such signals were observed in the amplified products of other fungal species (Figure 2b). Similarly, the results of the RPA-CRISPR/Cas12a-PLFS assay demonstrated evident red bands at the test line of the strip in the amplified products of the *S. terrestris* sample, contrasting with the control samples and other fungi where bands were solely observed at the control line (Figure 2c,d).

### 3.2. Optimization of RPA Reaction Time and Temperature

The amplified products from the RPA reactions with different reaction times, 10, 20, 25, 30, and 40 min, were analyzed by agarose gel electrophoresis. The results indicated that the intensity of the target bands increased with longer RPA amplification times (Figure 2e), while no distinct bands were observed in the negative controls. Brighter bands were observed in the agarose gel images for reaction times at 20–40 min. To meet the needs of detection effect and detection speed at the same time, a 20 min amplification time was selected as the experimental condition for the subsequent RPA-CRISPR/Cas12a detection system.

Furthermore, the amplified products from RPA reactions conducted at temperatures of 33, 35, 37, 39, and 41 °C were subjected to agarose gel electrophoresis analysis (Figure 2f). The results demonstrated the presence of target bands under all temperature conditions. The 39 °C temperature was selected as the experimental condition for the subsequent RPA-CRISPR/Cas12a detection system to ensure efficient detection.

### 3.3. Optimization of CRISPR/Cas12a-FRB Reaction Condition

**Figure 3:**
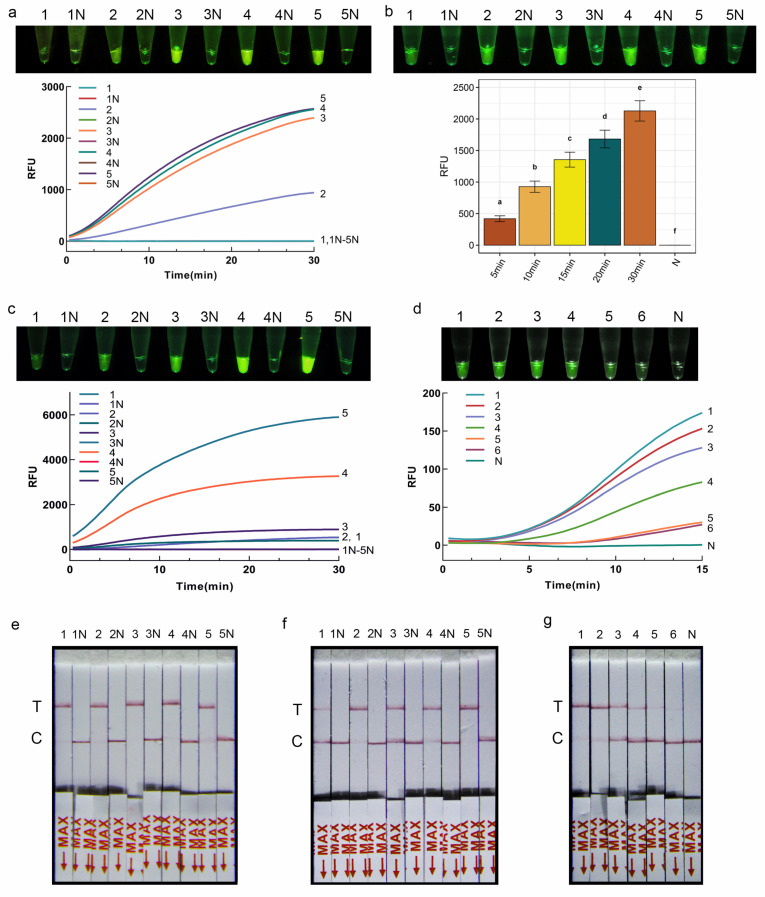
(**a**–**d**) RPA-CRISPR/Cas12a-FBR assay for *S. terrestris*. (**a**) Optimization of Cas12a/CrRNA concentrations for visual detection and their real-time fluorescent signals. 1–5: The ratio of Cas12a/crRNA: 0 nM/0 nM, 50 nM/100 nM, 100 nM/200 nM, 150 nM/250 nM, 200 nM/300 nM; 1N–5N: ddH_2_O. (**b**) Optimization of reaction time for visual detection and their real-time fluorescent signals. 1–5: 5 min, 10 min, 15 min, 20 min, 30 min; 1N–5N: ddH_2_O. The bar chart represents data obtained from three repeated experiments, different lowercase letters denote significant differences between the groups. (**c**) Optimization of FQ-DNA reporter molecule concentrations in the reaction system and their real-time fluorescent signals. 1–5: 50 nmol/L, 100 nmol/L, 200 nmol/L, 400 nmol/L, 800 nmol/L; 1N–5N: ddH_2_O. (**d**) Sensitivity verification of RPA-CRISPR/Cas12a-FBR visual detection system. 1–6: 1 ng, 0.1 ng, 10 pg, 1 pg, 0.1 pg, 0.01 pg. N: ddH_2_O. (**e**–**g**) RPA-CRISPR/Cas12a-PLFS assay for *S. terrestris*. (**e**) Optimization of LF-DNA reporter molecule concentrations. 1–5: 50 nmol/L, 100 nmol/L, 200 nmol/L, 400 nmol/L, 800 nmol/L; 1N–5N: ddH_2_O. (**f**) Optimization of reaction time for visual detection. 1–5: 5 min, 10 min, 15 min, 20 min, 30 min; 1N–5N: ddH_2_O. (**g**) Sensitivity verification of RPA-CRISPR/Cas12a-PLFS visual detection system. 1–6: 1 ng, 0.1 ng, 10 pg, 1 pg, 0.1 pg, 0.01 pg. N: ddH_2_O.

The CRISPR/Cas12a system, when combined with RPA, enhances the specificity and accuracy of molecular identification. The specific crRNA binding to the target sequence initiates the activation of Cas12a enzyme for the cleavage of the fluorescent probe, leading to the release of fluorescent signals. In this study, the amounts of Cas12a and crRNA required to induce significant green fluorescence were optimized, and the results showed that the reaction solution was colorless and transparent when the concentration ratio of Cas12a to crRNA was 0 nM/0 nM, and weak green fluorescence appeared when a ratio of 50 nM/100 nM was used, while an obvious fluorescence intensity was achieved at ratios of 100 nM/200 nM, 150 nM/250 nM, and 200 nM/300 nM (Figure 3a). Therefore, to ensure experimental results and save experimental costs at the same time, the ratio of 100 nM Cas12a to 200 nM crRNA was selected as the optimal reaction condition for subsequent experiments.

The optimization results of the fluorescent reporter molecule concentration indicated that when the final concentration of the FQ-DNA reporter molecule was 50 nmol/L, the CRISPR/Cas12a-FBR detection system exhibited a faint fluorescence signal (Figure 3c). As the concentration of the reporter molecule increased, the fluorescence intensity also increased accordingly. To balance cost considerations with fluorescence intensity, this study opted for a final concentration of 200 nmol/L for the fluorescent reporter molecule as the optimal choice (Figure 3c). Furthermore, the CRISPR/Cas12a-FBR was conducted with times ranging from 5 to 30 min, and a reaction time of as little as 5 min was enough to obtain a weak fluorescence signal (Figure 3b).

### 3.4. Optimization of CRISPR/Cas12a-PLFS Reaction Condition

Similar to the CRISPR/Cas12a-FRB detection system, the concentration of reporter molecule and reaction time of CRISPR/Cas12a-PLFS was optimized. Upon achieving a terminal concentration of 50 nmol/L for the LF-DNA reporter molecule, a conspicuous detection band became visible on the test strip (Figure 3e). Despite the detection of amplification products within a 5 min timeframe, the faintness of the test line suggested that an extended reaction period of 10 min was deemed optimal for enhanced sensitivity and accuracy (Figure 3f).

### 3.5. Sensitivity of RPA-CRISPR/Cas12a Assay

To assess the sensitivity of the RPA and RPA-CRISPR/Cas12a detection systems, varying concentrations of genomic DNA from *S. terrestris* were utilized. The findings indicated that a minimum of 1 ng DNA represented the detection limit for the RPA system, while the incorporation of the CRISPR/Cas12a detection system significantly improved the sensitivity of DNA detection. Examination using RPA-CRISPR/Cas12a-FRB revealed the detection of a faint fluorescence signal at a DNA concentration of 0.01 pg (Figure 3d). Similarly, in the RPA-CRISPR/Cas12a-PLFS experiments, a clear strong color signal was evident on the test line of the strip when the DNA concentration surpassed 1 pg. However, with a further reduction in DNA concentration to 0.01 pg, the test lines became blurred but remained distinguishable on the lateral flow dipsticks (Figure 3g). These outcomes affirm that the RPA-CRISPR/Cas12a assay is proficient in recognizing DNA concentrations as low as 0.01 pg/μL of *S. terrestris* DNA.

### 3.6. Application of RPA-CRISPR/Cas12a Assay for Plant Samples

**Figure 4:**
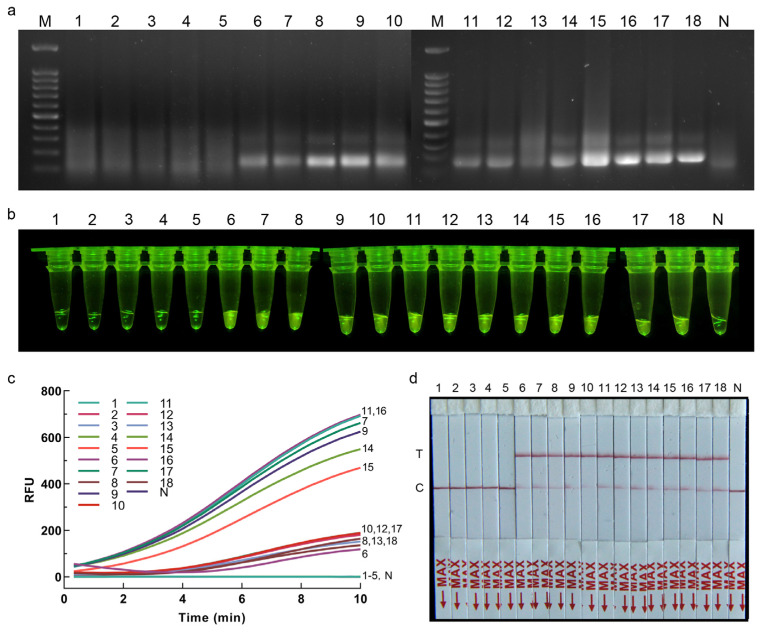
(**a**–**d**) Detection of plant samples using RPA-CRISPR/Cas12a assay. (**a**) RPA detection assay. (**b**) RPA-CRISPR/Cas12a-FRB. (**c**) Real-time fluorescent signals in RPA-CRISPR/Cas12a-FRB assay. (**d**) RPA-CRISPR/Cas12a-PLFS. 1–5: healthy onion samples, sample No. YCCK1, YCCK2, YCCK4, YCCK6, YCCK13, respectively; 6–17: inoculated onion samples with *S. terrestris*, sample No. YC4, YC6, YC8, YC9, YC11, YC13, YC14, YC15, YC17, YC18, YC19, YC20, respectively; 18: *S. terrestris*; N: ddH_2_O.

The feasibility of the developed RPA-CRISPR/Cas12a assay for infected plant samples was evaluated using inoculated onions. After 7 d of inoculation, pinkish-red discoloration was observed on the roots of the onions, whereas no symptoms were observed on the onions inoculated with sterile agar. The results of the RPA-CRISPR/Cas12a-FRB detection showed that *S. terrestris*-inoculated onion tissues and the positive control exhibited bright-green fluorescent signals, while no such signals were observed in the amplified products of healthy plant samples and the negative control (Figure 4b,c). Similarly, the results of agarose gel electrophoresis of RPA products and the RPA-CRISPR/Cas12a-PLFS assay were consistent with those of the RPA-CRISPR/Cas12a-FRB assay (Figure 4a,d).

## 4. Discussion

The increasing globalization of trade represents a significant threat due to the spread of invasive alien phytopathogens through various pathways, including cargo, passengers, conveyances, postal services, wooden packaging and containers [20]. *Setophoma terrestris*, a common soil resident, is responsible for causing pink root rot in multiple crops, including onion, muskmelon, watermelon, maize, squash, and canola plants [3,21,22,23,24]. The intensification of global trade has increased the risk of introducing *S. terrestris* into new regions, emphasizing the necessity for the efficient identification and management of this plant pathogen in order to safeguard agricultural interests. While Yoshida [25] established a SYBR Green qPCR assay for this pathogen, it is important to note that SYBR Green can bind non-specifically to the small groove of double-stranded DNA, leading to challenges in differentiating primer dimers or non-specific amplification products, and susceptibility to false positives and fluorescent signals being detected, often necessitating a combination with a melting curve analysis to exclude false positives. Likewise, in an effort to enhance the accuracy of the assay, Li et al. [11] established a TaqMan MGB-based qPCR. However, both detection assays have high equipment requirements and face challenges in meeting the demand for on-site rapid testing, presenting significant limitations. In contrast, the RPA operates within a stable temperature range of 37 to 42 °C, making it accessible through various means such as warm water, an incubator, room temperature, or even body heat. The compatibility of RPA with CRISPR-Cas12a detection is promising, given their congruent temperature requirements.

This study introduced two rapid and precise visual detection methods for *S. terrestris*, namely RPA-CRISPR/Cas12a-FRB and RPA-CRISPR/Cas12a-PLFS. The RPA-CRISPR/Cas12a-FRB approach can accurately detect *S. terrestris* within a 30 min timeframe, boasting a sensitivity of up to 0.01 pg/μL. Contrasted with the RPA-CRISPR/Cas12a-FRB system, the RPA-CRISPR/Cas12a-PLFS method can deliver precise results within 30 min, without the necessity of additional blue light equipment, featuring a sensitivity of 0.01 pg/μL. When considering quick inspections at customs, a rapid DNA extraction method can be used as an alternative before applying RPA-CRISPR/Cas12a assays, i.e., placing the hyphae in a centrifuge tube containing 50 μL of sterile ddH_2_O, heating it at 95 °C for 10 min, and then centrifuging to obtain the supernatant. If the rapid DNA extraction method mentioned in this study is employed, the entire detection process will not exceed 40 min, including DNA extraction, RPA reaction, Cas12a cleavage, and result readout.

In the RPA-CRISPR/Cas12a detection assay, we first evaluated the optimal RPA reaction time and temperature based on the gel electrophoresis band brightness of the RPA reaction product. This is mainly because the band brightness is related to the concentration of the amplification product, and the concentration directly affects the sensitivity of the CRISPR/Cas12a assay. In this study, we also tried to reduce the overall RPA-CRISPR/Cas12a detection process by shortening the RPA reaction time. However, when the RPA reaction time was shortened to 10 min, the sensitivity of the subsequent CRISPR/Cas12a visual detection method could only reach 0.1 ng. Therefore, the RPA reaction time of 20 min was chosen for this study in order to balance the detection effect and duration simultaneously.

When compared to real-time PCR, the developed RPA-CRISPR/Cas12a system offers simpler procedures, milder conditions, more adaptable signal readouts, and notably higher sensitivity, as the established minimal detection limit of the real-time PCR method for *S. terrestris* is 1 pg/uL [11]. Therefore, the visual detection approaches outlined in this study enable rapid and accurate screening for *S. terrestris* at ports and agricultural sites, mitigating the spread through the import, export, and transport of seeds and other reproductive materials. However, in comparison to real-time PCR, the RPA-CRISPR/Cas12a-FRB and RPA-CRISPR/Cas12a-PLFS methods present a disadvantage in terms of cost, with a price that is approximately 3–5 times higher than that of real-time PCR detection methods. This is primarily due to the relatively higher cost of enzymes. Nevertheless, it is anticipated that with the progression of technology, the expense of enzymes will be diminished in the future, rendering this method more convenient than real-time PCR.

The ITS sequence of *Setophoma* is distinctly different from that of other genera, ensuring specificity in primer design for this study. Therefore, strains from other genera were not used for verification during the experimental verification stage. Additionally, the total DNA of onion tissue also includes DNA from other microbial groups. The fact that healthy onion tissue did not yield positive results in the RPA-CRISPR/Cas12a assays further confirms the reliability of this study.

Nonetheless, we acknowledge that this study has certain limitations. One of the important issues is the restricted number and diversity of *S. terrestris* samples included in this study for testing primers, largely due to challenges in accessing materials for studying imported quarantine fungi in China. Despite this limitation, our sequence similarity comparisons and phylogenetic analyses indicate distinct ITS region sequence differences between *S. terrestris* and its close relatives. Furthermore, ITS sequences from strains originating from different countries show relative conservation. Therefore, we are confident that the primers developed in this study are specific and expect to further validate the validity and sensitivity of the RPA-CRISPR/Cas12a assay through international collaboration. In addition, as *S. terrestris* is a quarantine fungus for entry into China, no diseased plants infected by this species have been found in the field. Consequently, it is not currently possible to conduct tests on real field-infested material. However, the tests conducted using artificially inoculated onion tissues provide evidence that the detection method developed in this study is feasible.

## Figures and Tables

**Table 1:** Fungal strains used in this study.

Fungal Name	Strain Number ^1^	Type	Substrate	Location	ITS GenBank Accession	Reference
*Setophoma antiqua*	LC6596	ex-holotype	*Camellia sinensis*	China	MK525001	Liu et al. (2019) [10]
*Setophoma caverna*	LC12841	/	Carbonatite	China	MK525016	Crous et al. (2019) [18]
*Setophoma endophytica*	LC3163	ex-holotype	*Camellia sinensis*	China	MK511931	Liu et al. (2019) [10]
*Setophoma endophytica*	LC3164	/	*Camellia sinensis*	China	MK511932	Liu et al. (2019) [10]
*Setophoma longinqua*	LC6593	ex-holotype	*Camellia sinensis*	China	MK511908	Liu et al. (2019) [10]
*Setophoma terrestris*	CBS 335.29	ex-holotype	*Allium sativum*	North America	KF251246	Liu et al. (2019) [10]
*Setophoma terrestris*	CBS 122483	/	*Allium cepa*	Vietnam	/	/
*Setophoma terrestris*	CBS 335.87 *	/	*Allium cepa*	Senegal	KF251247	Liu et al. (2019) [10]
*Setophoma terrestris*	CBS 377.52 *	/	*Allium cepa*	Unknown	KF251248	Liu et al. (2019) [10]
*Setophoma yunnanensis*	LC6753	ex-holotype	*Camellia sinensis*	China	MK511913	Liu et al. (2019) [10]
*Setophoma yingyisheniae*	LC13479	ex-holotype	*Camellia sinensis*	China	MK525007	Liu et al. (2019) [10]

Note: ^1,^* only used for primer designation, while the others used for molecular diagnosis.

**Table 2:** Primer, guide RNA and reporter used in this study.

Primer Name	Sequence (5′-3′)	Length	Objective
St-RPA-F	5′-GGGCGTCTTGTCGTATTACGACTCGCCTTAAATT-3′	34	RPA
St-RPA-R	5′-CCCTACCTGATCCGAGGTCAAACGTGGTAAATGT-3′	34	
St-crRNA	5′-UAAUUUCUACUAAGUGUAGAUCGAUCGUAGCCCGUUGUACUGG-3′	43	
FQ-DNA	5′-(FAM) TTATT (BHQ1)-3′	5	CRISPR/Cas12a-FRB
LF-DNA	5′-(FAM) TTTTTTTTTT (Biotin)-3′	10	CRISPR/Cas12a-PLFS

## Data Availability

The original contributions presented in the study are included in the article, further inquiries can be directed to the corresponding author.
